# Gamifying Cognitive Behavioral Therapy Techniques on Smartphones for Bangkok’s Millennials With Depressive Symptoms: Interdisciplinary Game Development

**DOI:** 10.2196/41638

**Published:** 2023-05-12

**Authors:** Poe Sriwatanathamma, Veerawat Sirivesmas, Sone Simatrang, Nobonita Himani Bhowmik

**Affiliations:** 1 Doctor of Philosophy Program in Design Arts (International Program), Faculty of Decorative Arts Silpakorn University Bangkok Thailand; 2 Department of Game Design New York University New York, NY United States

**Keywords:** cognitive behavioral therapy, gamification, Bangkok’s millennials, depressive symptoms, mobile phone

## Abstract

**Background:**

There is serious concern over the annual increase in depressive symptoms among millennials in Bangkok, Thailand. Their daily routine revolves around the use of their smartphones for work and leisure. Although accessibility to mental health care is expanding, it cannot keep up with the demand for mental health treatment. Outside Thailand, multiple projects and studies have attempted to merge gamification mechanisms and cognitive behavioral therapy (CBT) to create mobile health intervention apps and serious games with positive feedback. This presents an opportunity to explore the same approach in Thailand.

**Objective:**

This study investigated the development process of gamifying CBT techniques to support game mechanics in a visual narrative serious game, BlueLine. The primary target of this research is Bangkok’s millennials. In the game, players play as Blue, a Bangkok millennial who struggles to live through societal norms that influence his digital life and relationships. Through in-game scenarios, players will learn and understand how to lessen the impact of depressive symptoms via gamified interactions on their smartphones.

**Methods:**

First, this paper follows each development step of solidifying BlueLine’s game structure by integrating the Activating Events, Beliefs, Consequences, Disputation of Beliefs and Effective New Approaches (ABCDE) model and narrative in games. Second, the approach to select CBT and related therapeutic elements for gamification is based on suitability to the game structure. Throughout the process, CBT experts in Thailand have reviewed these scenarios. The approach forms the base of the player’s interactions throughout the scenarios in BlueLine, broken down into 4 types of gamified mechanisms: narrative, verbal interactions, physical interactions, and social media interactions.

**Results:**

With the game structure based on the ABCDE model, BlueLine scenarios implement gamified mechanisms in conjunction with the following CBT and related therapeutic elements: behavioral activation, self-monitoring, interpersonal skills, positive psychology, relaxation and mindful activities, and problem-solving. In each scenario, players guide Blue to overcome his triggered dysfunctional beliefs. During this process, players can learn and understand how to lessen the impact of depressive symptoms through gamified interactions.

**Conclusions:**

This paper presents the development process of gamifying CBT and related therapeutic techniques in BlueLine game scenarios. A scenario can harbor multiple techniques, including behavioral activation, self-monitoring, interpersonal skills, positive psychology, relaxation and mindful activities, and problem-solving. BlueLine’s game structure does not limit the fact that the same combination of CBT elements ties each gamified mechanism.

## Introduction

### Background

Nowadays, most millennials in Bangkok, Thailand, have adopted a long-hours working culture, which allows little time to relax and unwind. Social media has become the platform of choice, whether it is used to socialize with others or to catch up on public health warnings, such as those issued during the COVID-19 pandemic [1]. From 2016 to 2020, the number of social media users in Thailand jumped from 32 million to 52 million, approximately 80% of the population, with an average of 2 hours and 55 minutes of web-based social exposure per day [2]. After a long period of social media use, a possible side effect is social comparison, such as seeking acceptance and confidence from external sources [3]. Thus, it plays an essential role in influencing one to develop depressive symptoms [4].

A total of 20,685 Bangkok millennials responded to Thailand’s Department of Mental Health survey in 2021 [5]. The risk of developing depressive symptoms in this group is frighteningly high at 48.97%, and, compared with provinces outside the capital, it is 4 times higher [6]. The survey was completed using the Patient Health Questionnaire (2-question version, 8-question version, and 9-question version) mental health survey system that is based on the Diagnostic and Statistical Manual of Mental Disorders, Fifth Edition (DSM-5).

Since the outbreak of COVID-19, depressive symptoms have become more apparent among Bangkok’s millennials [5-7]. During the pandemic, there were constant actions to promote mental health care awareness by hospitals and schools to provide quality and sustainable mental health care services [8]. Nonetheless, exposure to help is still limited, as was the case especially during the COVID-19–related lockdown period [9,10]. More services must therefore explore other approaches to reach patients experiencing depressive symptoms.

Smartphones are becoming a unanimous part of our daily routine, creating a window of opportunity to increase access to mental health care [11]. This can be in the form of serious games (designed for purposes beyond entertainment), which are part of applied games; games based on cognitive behavioral therapy (CBT) constitute 1 of the 6 major categories of tested games for mental health [12]. This approach can benefit players with favorable emotional treatment through awareness, behavior changes, and relief of symptoms [13,14]. Mobile mental health intervention is affordable and can be used adjunctively with the patient’s existing treatment [15].

### Integrating CBT Techniques Into Games

In recent years, studies have attempted to develop frameworks to create practical approaches to developing gamified apps. However, most still need to be fully established [16,17]. It is challenging to integrate CBT into different contexts of each game while ensuring that it achieves the intended experience and treatment goal for the players [18]. In this paper, the term *intended experience* refers to the expected gameplay experience of the player (ie, the quality of the gameplay interactions) [19]. It is plausible to integrate CBT techniques into games as multiple successful projects existing in different regions have shown (eg, a mobile health [mHealth] app for Māori [Indigenous New Zealand people] youth with emotional difficulties, Quest Te Whitianga) [20]. Night in the Woods (a winner of the British Academy of Film and Television Arts games award for narrative) is the story of Mae, who is dealing with depression and anxiety as she quietly returns to her hometown [21].

This paper breaks down the development process of a gamified intervention incorporating CBT techniques into BlueLine. The overall intended experience for BlueLine is for the player to live through scenarios as the main character where they are given choices and can perform actions that incorporate the following therapeutic components: behavioral activation, self-monitoring, interpersonal skills, positive psychology, relaxation and mindful activities, and problem-solving.

## Methods

### Overview

BlueLine is a visual narrative serious game where the player plays as Blue, a Bangkok millennial who struggles to live through societal norms that influence his virtual life and relationships. The game is inspired by Florence [22], in terms of the way the narrative expresses through visuals, and by We Should Talk [23], in terms of how the player’s verbal choices affect their emotional state and lead to certain consequences in the game’s world. This game primarily targets mobile platforms, and most Bangkok millennials use them, especially those who exhibit depressive symptoms. The game is also designed to be approachable to casual players. On release, BlueLine will be playable in both Thai and English to support the primary target group.

In the early stages of developing BlueLine, the focus was to create prototypes that could successfully integrate the gamification of CBT techniques into narrative design and harmoniously use visual design as the core gameplay. Our design process includes medical doctors and therapists who frequently care for Bangkok’s millennials. It allows us to circumvent the limited studies on the use of CBT in Thai games available at the time of this research. We will conduct a randomized controlled trial of the complete BlueLine prototype to assess its efficacy, acceptability, and usability with the support of Thailand’s Department of Mental Health.

First, we must understand the behavior of our primary target audience, the millennials of Bangkok. The behavior patterns of popular activities among this group establish statistics, especially the popular interactions in each social media application [24,25]. It also includes the time spent on each web-based activity. From a game design point of view, creating scenarios by researching and understanding the habits of the target audience can lead to better engagement and enable delivering of the intended experience [26]. Our design team has created these scenarios to be approachable even to people who do not have depressive symptoms. Through these CBT-gamified interactions, players can absorb information to become familiar with how to cope with depressive symptoms. They can fail safely without social consequences in real life. Each game scenario will also have its intended experience, aligning with its intended experience.

Second, we aimed to obtain insight from experts who have experience using CBT techniques to treat Bangkok’s millennials. For a better understanding of the game design of this project, we created short scenarios in the form of storyboards and gameplay mock-ups in the form of animation to help the experts to express their opinion on how the game will affect their patients and to suggest details that can improve the effectiveness of the CBT-infused interactions. These short scenarios are experimental prototypes that explore how we can merge CBT techniques and gamification elements. Successful prototypes that deliver the intended gameplay experience will be compiled into the game later.

The feedback from monthly meetings with the experts is critical in developing future iterations. Playtests were held within the team frequently, followed by group discussions, which were also immensely invaluable for our developers with CBT-related experience. They shared opinions on aspects that they feel are beneficial and need improvement. This technique of verbalizing thought processes in usability testing is called *thinking aloud* [27]. At this stage, the emphasis was on ensuring that the prototype was complying with the intended experience while incorporating placeholder art assets. This was a standard game development process to prove that the participative elements in the gameplay can be practical without having to rely heavily on aesthetics.

### Selecting CBT Techniques as the Game Structure

CBT is an evidence-based treatment for psychiatric disorders, including depressive symptoms. This treatment involves attempts to change ways of thinking and behavior patterns through understanding negative thoughts, emotions, behaviors, and physiological reactions, thereby relieving their symptoms [28]. This helps individuals learn to be their own therapists [29].

For BlueLine, the game’s core structure implements the Activating Events, Beliefs, Consequences, Disputation of Beliefs and Effective New Approaches (ABCDE) model. It is a simple mnemonic developed in the field of rational-emotive behavior therapy by Albert Ellis [30]. This model targets beliefs as a fundamental course of treatment. It guides the individual to break down the event and carefully examine the causes and effects so that they may respond to the situation in an improved manner if it recurs [31].

The core structure often integrates the ABCDE model in several CBT-based applications and serious games. One such application is Self-help, Integrated, and Gamified Mobile Phone Application, a gamified mHealth intervention, and it is used as an evidence-based theoretical structure for players to overcome their maladaptive beliefs [32], whereas REThink is a clinically tested therapeutic game for college students to manage distress efficiently [33], and ReWIND is a role-playing game that allows players to overcome their generalized anxiety disorder through anxiety-causing encounters [34]. Similar to BlueLine, they all use the ABCDE model to challenge dysfunctional beliefs in a safe virtual environment for their audience. Following this model, BlueLine’s structure is divided into 5 steps (Textbox 1).

These 5 steps can match well with the narrative in a visual narrative game structure, as shown in Textbox 2. We can resolve the narrative tension by disputing beliefs and practicing new approaches. In addition, this allows other CBT and related therapeutic techniques to work in conjunction and still perform efficiently, including behavioral activation [35], self-monitoring [36], interpersonal skills [37], positive psychology [38], relaxation and mindful activities [39], and problem-solving [40].

**The Activating Events, Beliefs, Consequences, Disputation of Beliefs and Effective New Approaches (ABCDE) model as a game structure from a player’s point of view.**

**A: activating event**

- Players identify their main character’s trigger during a distressing scenario in the game.

**B: beliefs**

- Players understand the underlying dysfunctional belief as the scenario plays out by observing how the main character reacts.

**C: consequences**

- The impacts of the main character’s beliefs influence physically within the game’s world (character actions and surroundings) and psychologically (emotions).

**D: disputation of beliefs**

- The main character will attempt to challenge their dysfunctional beliefs through a series of player interactions in the game.

**E: effective new approach**

- The main character manages to transform their initial dysfunctional beliefs. The player will witness positive changes in the main character’s mindset.

**A narrative scenario in the Activating Events, Beliefs, Consequences, Disputation of Beliefs and Effective New Approaches (ABCDE) model.**

**Activating event**

- Blue is forced to stay late at the office. Even after he gets home, he gets another call from work and has to stay up to work.

**Beliefs**

- He feels worthless. No matter how hard he works, the office is not happy.

**Consequences**

- He feels unappreciated, drained owing to lack of sleep, and loses interest in activities.

**Disputation of beliefs**

- He recognizes that the office is taking advantage of him and that he should not devalue his worth based on other people’s words.

**Effective new approach**

- He discusses with the management his plan to not work after business hours and starts to perform recreational activities instead.

BlueLine simulates scenarios in safe virtual environments; players have the comfort of time to identify dysfunctional beliefs before they can happen. This way, they can see the consequences of their choices through self-monitoring the game character. The game allows players to challenge dysfunctional beliefs through its main character, Blue.

Following this structure, we aimed to scale up BlueLine because we plan the gameplay length to be approximately 1 hour and 20 minutes. We aimed to manage players’ time on a single playthrough of BlueLine, thus lowering the risk of preoccupation with the game, which is one of the symptoms of internet gaming disorder in DSM-5, Text Revision [41]. Several factors are related to addictions, such as life satisfaction [42] and the types of games (on the web and offline) [43]. Statistically speaking, players who spend long weekly gaming hours (approximately 20 hours per week) are more likely to develop depressive and addiction symptoms [44].

BlueLine gameplay has 15 chapters, each lasting 5 minutes on average. The game will offer pause and resume features. Players do not have to finish a chapter in 1 session and have the option to resume from where they left off. Our players may not return to the game because a lack of motivation can be related to depressive symptoms [45]. This presents an opportunity for a reminder feature to encourage players to resume the game through timely notifications; for example, the game would deliver notifications during the evening but not late at night. This reminder feature also exists in internet-based CBT applications [46] and eHealth programs [47].

Storyboards are also developed during this process, with mock-ups created to test the composition of visual elements on a smartphone screen. While sketching the storyboards, many discussions arose about specific moments, leading to more inspiration to develop the narrative further. We can determine which CBT-infused action the main character could perform by seeing the sketched character in the scenario. We specifically designed the color palette and animation to ensure that players are not exposed to specific light patterns or flashing lights, which can lead to photosensitivity epilepsy or blackouts. Thus, storyboards guide the next step—the gamification of CBT techniques.

### Approach to Gamification of CBT

The challenge is to gamify CBT techniques appropriately and fit them into each scenario. The chosen approach is based on case-by-case scenarios, with the end goal being that the interactions should be meaningful to the player. Meaningful play occurs when the player’s action has immediate importance and affects the larger context of the game [48]. We were able to accomplish this by basing the content of BlueLine on Bangkok’s millennials, including their daily routines, behaviors, and life experiences. This shapes the identity of Blue (the main character), who would face and overcome obstacles in the story.

### Gamification Elements

We divide BlueLine’s CBT gamification elements into 4 main components: narrative, verbal interactions, physical interactions, and social media interactions. We acknowledge that existing studies discuss gamification elements in CBT-based applications, such as scores, levels, and tokenized rewards [49,50]. However, there are difficulties implementing them in BlueLine because it is not in the nature of a visual narrative game to have repetitive elements. Table 1 shows the core structure of gamification mechanisms, and other use cases will be discussed in the *Results* section.

**Table 1:** Gamified mechanisms in BlueLine.

Gamification of CBT^a^ in mechanics and description		Use in BlueLine
**Narrative**		
	Growth of Blue as a representation of Bangkok’s millennials	The story is told from the perspective of Blue and his millennial daily routine, lifestyle, and life choices. Through the ups and downs in the story, Blue struggles with the triggering of his dysfunctional beliefs and overcoming them. CBT techniques: self-monitoring, problem-solving, and interpersonal skills
**Verbal interactions**		
	Engagement in conversation	Blue participates in conversations with NPCs^b^ throughout the game. Each NPC’s reply contains therapeutic components depending on the player’s verbal choices. CBT techniques: self-monitoring, interpersonal skills, and behavioral activation
**Physical interactions**		
	Activities that require Blue’s physical action	Blue participates in multiple activities that require him to perform the task physically. Interactions range from basic actions such as walking to complicated tasks such as grocery shopping and giving a love letter. CBT techniques: relaxation and mindful activities and problem-solving
**Social media interactions**		
	Scrolling through social media and likes	This feature mimics a social app where players can scroll through the social feed in the game’s world. The content in the feed is generated by design to match the intended experience of that scenario. It is not linked to any actual social apps. Generated posts are visual based to avoid negative trigger words. CBT technique: self-monitoring
	Photo mode	This feature is a version of the camera app on mobile phones. The scenes that players see through the lens are generated by design, inspired by actual real-life locations. CBT techniques: relaxation and mindful activities

^a^CBT: cognitive behavioral therapy.

^b^NPC: nonplayer character.

## Results

### Overview

This section details several scenarios where each gamification mechanism is integrated with CBT and therapeutic techniques. There is a degree of flexibility in which specific scenarios can have multiple gamification mechanisms and therapeutic techniques, whereas the core structure includes the ABCDE model.

### Narrative

Depressive symptoms can recur, and that is the takeaway from BlueLine, which shows through player interactions that CBT and therapeutic techniques can reduce the risk of relapse as long as it is maintained [51]. BlueLine will take players through multiple scenarios built under the expanded ABCDE structure (Tables 2 and 3). This version adds a chapter column to support the upscaled story. A DSM-5 column is added to assess Blue’s depressive symptoms at each stage of the story. This serves 2 purposes. First, it keeps the developers and consulting experts informed about exposure to depressive symptoms during the design and production stages. Second, it identifies moments in BlueLine where the game could encourage players to be open to seeking mental health care. This approach could circumvent the unwillingness of Asian communities to access help from mainstream services [52]. Throughout the game, visual keys are placed to subtly help the player gain confidence to reach out for help if needed; for example, a customized billboard of a real-life mental health care service will be placed far in the background but still legibly visible.

**Table 2:** BlueLine’s Activating Events, Beliefs, Consequences, Disputation of Beliefs and Effective New Approaches (ABCDE) structure with a narrative framework and a Diagnostic and Statistical Manual of Mental Disorders, Fifth Edition (DSM-5) column.

BlueLine: Chapter 9, scenario 3	DSM-5	ABCDE model
	A1^a^-A9	
Blue and his partner have a minor quarrel over the photos he has taken.	—^b^	Activating event: Blue tries to apologize because the photos he took were not his best.
Line thinks that Blue is not trying hard enough and believes that he does not care.	—	Beliefs: Blue feels he has messed up. In the hope of being forgiven by Line, he keeps apologizing to her.
Line vents at Blue.	—	Consequences: Blue feels insignificant and worthless because she despises the photos he took.
The player stops Blue from panicking.	—	Disputations of belief: Blue stops apologizing (stays silent) so that Line can gather her thoughts.
Line realizes that Blue did try his best.	—	Effective new approach: Blue learns that by not panicking, he enables Line to understand his worth.

^a^A1 to A9: assessment of depressive symptoms at each stage of the story.

^b^To be filled in by the developers and consulting experts.

**Table 3:** An ABCDE^a^ model breakdown of the conversation exchanged between Blue and Line in chapter 9 of BlueLine.

	Activating event	Beliefs	Consequences	Disputation of beliefs	Effective new approach
Narrative	Blue and Line are having breakfast after the sunrise photo shoot. The couple begins to quarrel.	Line thinks that Blue is not trying hard enough and believes that he does not care.	Line vents at Blue.	Blue cannot continue to apologize because the game prevents the player from saying “Sorry” by fading out the “Sorry” button.	Line realizes that Blue does try his best and that the way she expresses herself has a negative effect on Blue.
Blue	Blue tries to apologize because the photos he took were not his best. Player begins self-monitoring.	Blue feels he has messed up. In the hope of being forgiven, he keeps apologizing (achieved by the player pressing the “Sorry” button). DSM-5^b^ symptoms: A7^c^: a sense of worthlessness A8: impaired concentration	Blue feels insignificant and worthless because she despises the photos. DSM-5 symptoms: A1: depressed mood A2: lack of interest A6: fatigue A7: a sense of worthlessness A8: impaired concentration	Interpersonal skills (using silence): Blue uses silence so that Line can gather her thoughts.	Behavioral activation: Blue learns that sometimes a moment of silence can help others to regain their minds.
Line	Line is angry because the photos taken by Blue are not good enough.	She thinks that the photos are bad because Blue does not care. She does not want to listen to any excuses or apologies.	Line feels unappreciated and blames Blue. DSM-5 symptoms A2: lack of interest A8: impaired concentration	Interpersonal skills (giving recognition): Line realizes that her words have hurt Blue.	Behavioral activation: Line learns to appreciate and give recognition when others are trying their best.

^a^ABCDE: Activating Events, Beliefs, Consequences, Disputation of Beliefs and Effective New Approaches.

^b^DSM-5: Diagnostic and Statistical Manual of Mental Disorders, Fifth Edition.

^c^A7 and so on: assessment of depressive symptoms at each stage of the story.

### Act 1: Refresh

It is a new beginning for Blue, who has just returned to Thailand to start a new chapter in his life with a good office job and to be with family. The player will begin appreciating the sense of well-being radiated by a motivated office worker through self-monitoring Blue’s daily routine. Blue will encourage the player to complete relaxation and mindful activities throughout the day by rewarding them with positive outcomes. This is achieved by engaging in physical interactions that some might consider mundane tasks (eg, waking up at 5 AM to leave for work early to avoid the traffic). At the same time, Blue will show appreciation for a beautiful sunrise. Eventually, the same routine begins to gray out as his surroundings slowly pressure him, such as being asked to work overtime. This will subtly trigger the activating event of the ABCDE model. The player’s interactions at this stage consist of following Blue’s attempts to find his way back to the well-being he once experienced.

### Act 2: Reaching Out

After unsuccessful attempts at socializing, Blue meets Line, a girl who is an office worker just like him. They start to bond and become more and more close after each meeting via verbal interactions. Line’s characteristics serve multiple vital purposes in the game. She serves as a source for Blue’s development; at times, her actions and speech are similar to those of a therapist (to help reduce the anxiety the player might feel regarding their first therapeutic appointment). When Blue is feeling low, she helps him by performing therapeutic-infused actions (eg, using interpersonal skills). She also encourages Blue to participate in relaxation and mindful activities to reduce his negative emotions via positive psychology [38] (eg, visiting gardens and mountains). After some time, she will trigger a dysfunctional belief stemming from upward social comparison. This will allow Blue and the player to reciprocate her kindness and problem-solving proficiency using the therapeutic techniques they learn during the game.

### Act 3: Reconnect

This act is set during the time frame of the 2020 COVID-19 pandemic in Thailand. Although the world changes, we want to present ways to handle mental health for our stakeholders. In the story, Blue’s parents ask him to return home during quarantine. He is hesitant to leave Line’s side because he only recently moved in with her, but she is supportive of his decision to go back home to take care of his parents. Slowly, however, distance begins to chip away at their bond. Depressive symptoms arise from activating events through social comparison in topics such as “It is safe to go on trips because there is no close contact if nobody goes on a trip,” even as the lockdown intensifies in Thailand. Ultimately, the two work together to dispute their different dysfunctional beliefs to save their relationship. The last problem-solving session involves using the related CBT techniques throughout the game to unfold the final problem. The game rewards the player with a fulfilling climax—narrative, gameplay, visuals, and sound at their peak—making the final interaction memorable for players so that they may recall the way they used behavioral activation to handle the situation were it to happen in real life.

### Verbal Interactions

**Figure 1:**
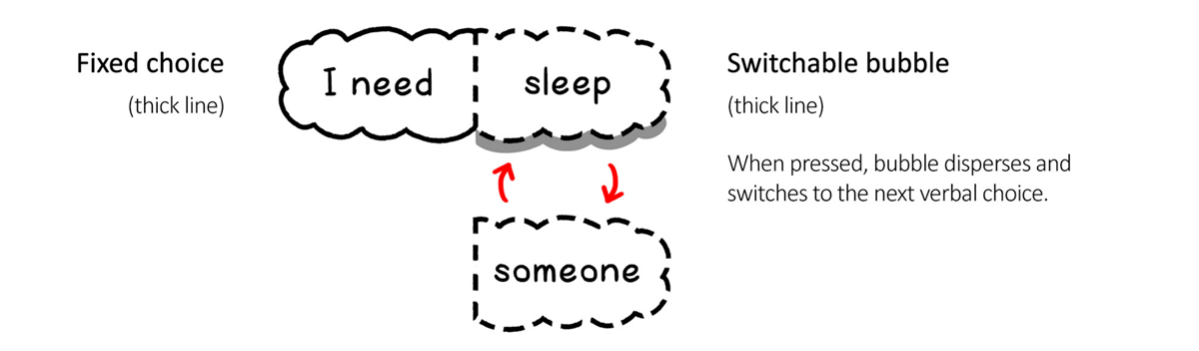
Dialogue-crafting system: single-choice sentence crafter.

Interactions with other nonplayer characters (NPCs) are essential in this game. Through their voices and actions, the NPCs aim to provide therapeutic components and gamification strategies, such as reinforcement of successful behavior, and provide feedback on the player’s performance through prompts [53]. Players can make verbal decisions through the dialogue-crafting system (Figure 1).

The dialogue-crafting system is inspired by the sentence-spinner mechanic from the game *We Should Talk*. The game’s use of sentence crafting creates different reactions. It focuses on using a designated combination of verbal choices for players to string together. In BlueLine, we have set up 2 types of sentence crafters: the single-choice sentence crafter allows the player to change 1 part of the sentence, whereas the dual-choice sentence crafter allows 2 parts to be changed.

**Figure 2:**
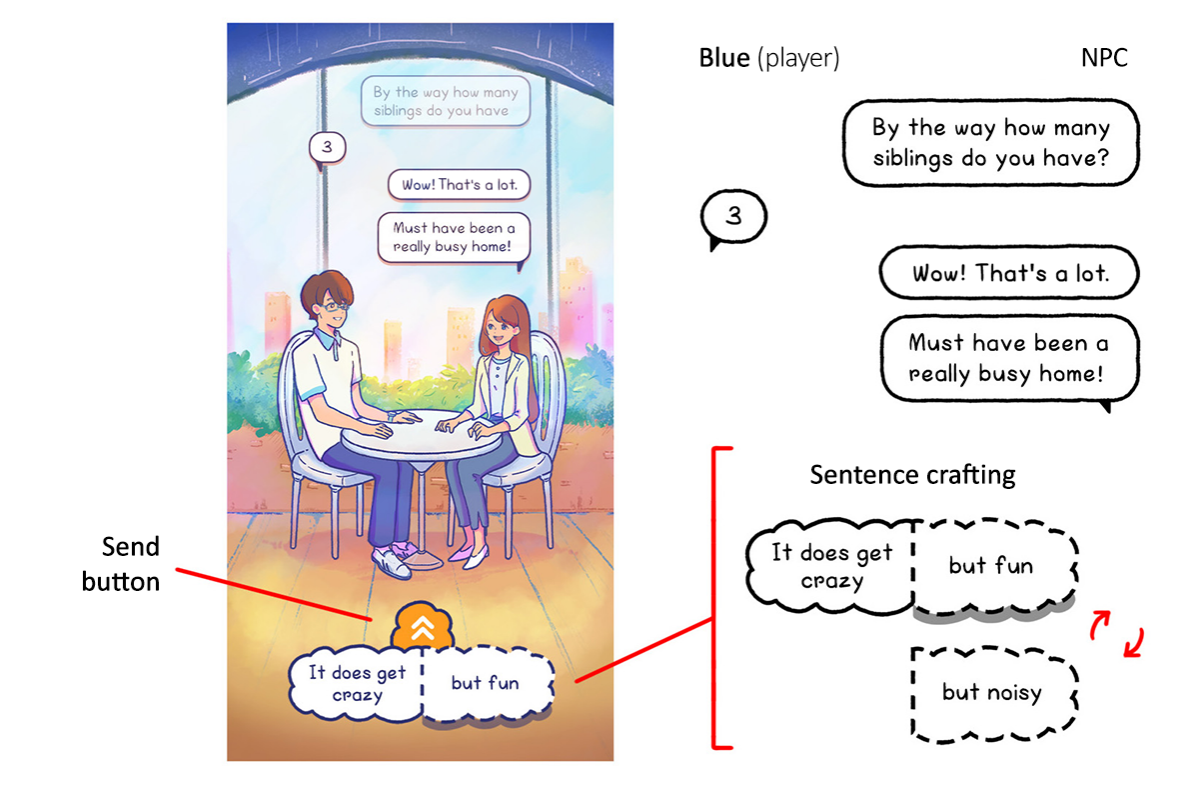
Breakdown of BlueLine’s single-choice sentence spinner.

CBT components will be integrated based on the verbal combination put together by the player and the feedback provided through the NPC, Line (Blue’s partner); for example, as shown in Figure 2, this conversation aims to surround the player with a therapeutic interpersonal relationship to make them feel cared for in a supportive, nonjudgmental environment [54].

The gameplay screen shows Blue (the player) and Line (the NPC) at a cafe. Their conversation is shown in the bubbles positioned above their heads. At the bottom is the sentence crafter. The phrase “It does get crazy” is prefixed, as shown by the solid line, whereas the switchable portion is designated by a dotted line. Although the 2 choices are worded differently, they have the same underlying intention, which is to create positive emotions through evoking a sense of well-being [38]; for example, a player whose childhood was filled with joyous and noisy moments will be able to relate more to these good memories than players whose childhood was not as joyous and noisy. This follows the concept of piggybacking in game design, using what players already know instead of teaching them from scratch [55].

The example presented in Table 3 involves depressive symptoms experienced by both Blue and Line. Together, they review the photos Blue took. Line does not like the photos; hence, Blue tries to apologize to her in an attempt to calm her down. A “Sorry” chat bubble will appear when the player presses the “Sorry” button at the bottom of the screen. However, no matter how often the player presses the “Sorry” button, Line does not calm down. To resolve this issue, the player has to stop pressing the “Sorry” button. This would prevent Blue from apologizing and enable Line to gather her thoughts. Ultimately, Line realizes that her words hurt Blue; she appreciates this and says, “Sorry. Thank you for trying your best.” Throughout this scenario, self-monitoring, interpersonal skills, and behavioral activation are used.

### Physical Interactions

In BlueLine, physical activities expose players to therapeutic components through actions. This chapter presents 2 examples: one concerns implementing art therapy as relaxation and mindful activities, and the other concerns problem-solving.

**Figure 3:**
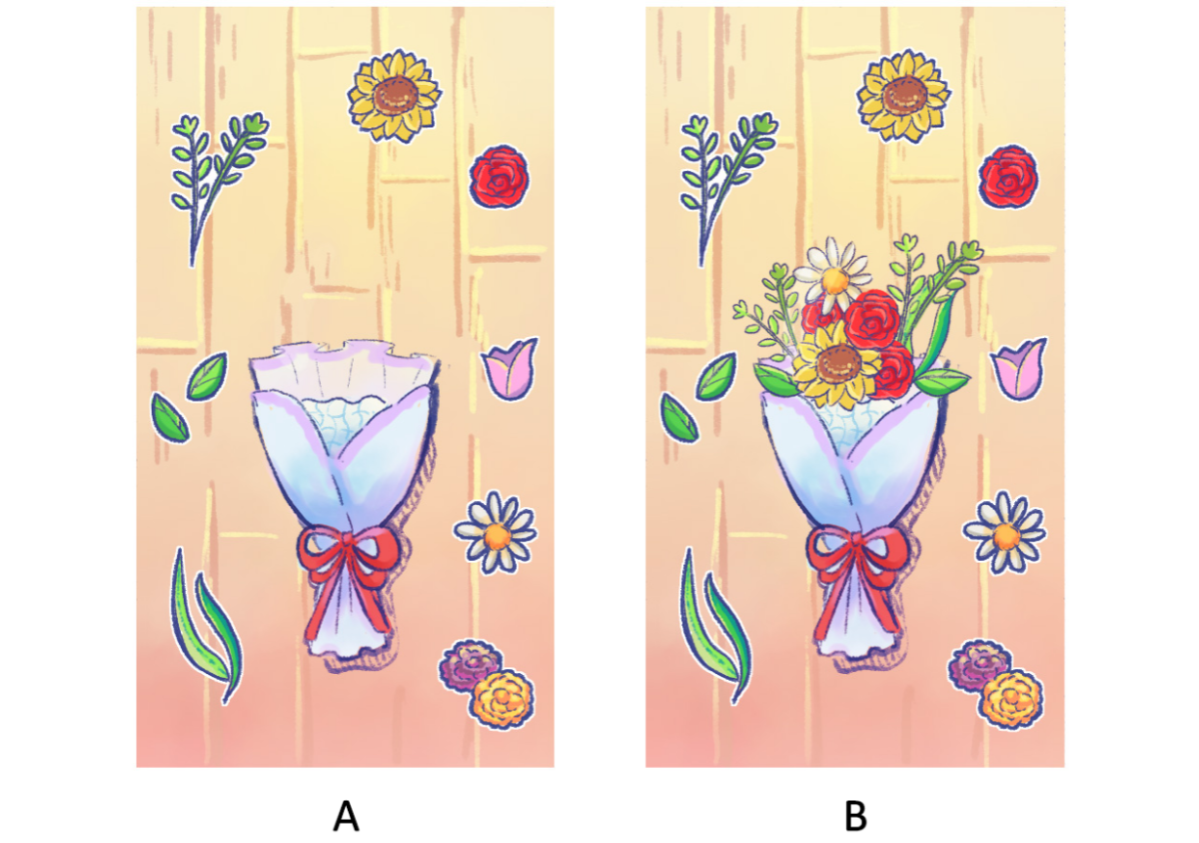
BlueLine’s handmade flower bouquet: (A) before gameplay and (B) after gameplay.

The first scenario occurs when Blue stops by to purchase a bouquet of flowers on Valentine’s Day. Instead of buying a premade bouquet, he makes one by hand (Figure 3). The goal is to teach the player that socializing is not the only means of engaging in relaxation and mindful activities. The process of art creation in art therapy also includes therapeutic elements such as self-exploration, which promotes an individual’s self-awareness in terms of seeing a more positive self-image [56]. Art creation is not tied to a single medium and can vary depending on the culture concerned; some examples are painting, music, and knitting [57,58].

To make the experience feel tangible, the player can decorate the bouquet using their fingers to drag and drop each flower. There is a minimum placement limit before the game can proceed to the next scenario, but the player can take their time until they want to move on. We consider not tying in reward-centric game elements, such as keeping score or points for tokenized rewards, and instead allowing the journey of the interactions itself to be the reward. From our experience, this helps player engagement to last longer.

There is a moment in act 3 when Blue goes to a supermarket during the 2020 COVID-19–related lockdown in Thailand. He picks up face masks, alcohol, tissue rolls, and other necessities, but only so many items are available on the shelves.

**Figure 4:**
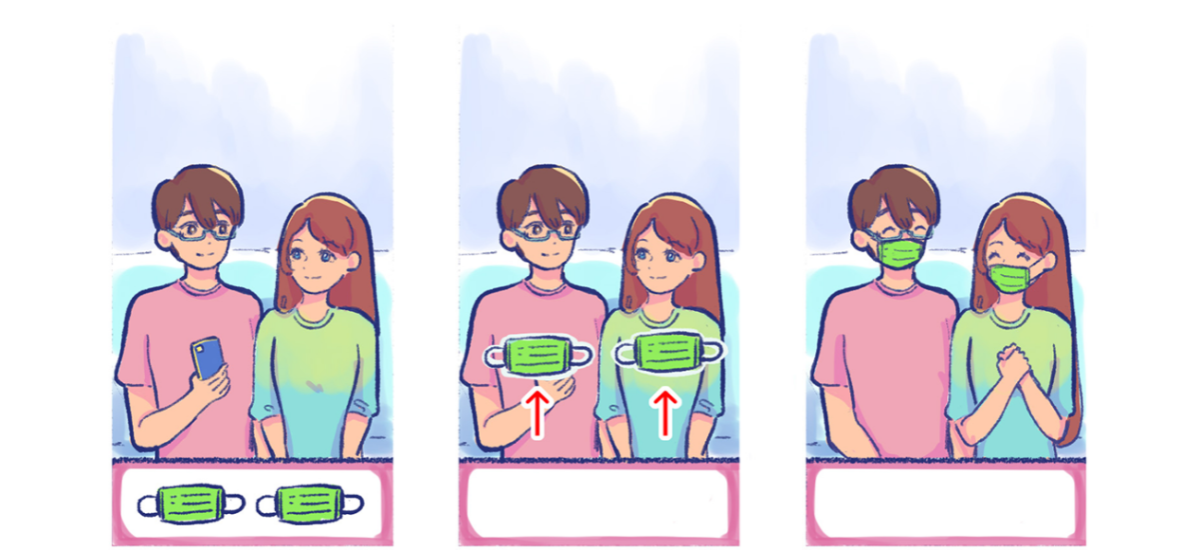
Wearing face masks during the COVID-19 pandemic.

The scenario at this stage of the game displays the risk of developing depressive symptoms during the COVID-19 pandemic in Thailand. As the population experiences more exposure to the disease and tunes in to information about COVID-19 on social media for at least 3 hours per day, there are more substantial effects on mental health in the form of anxiety and insomnia [7]. However, the process of problem-solving can be positively oriented. The solvable problem is set up for the player as a challenge and encourages their ability to resolve it effectively [40]. In Figure 4, the player will perform a swipe-up gesture with their finger to get both Blue (the player’s character) and Line (the NPC) to wear masks. The players can see the progress through visually animated feedback from the 2 characters.

### Social Media Interactions

According to the 2020 user behavior survey conducted by the Electronic Transactions Development Agency [59], Thai individuals spend 11 hours and 25 minutes on the internet daily, which is triple the amount compared with 2013. Instead of avoiding this topic, BlueLine uses it to visually compare a web-based lifestyle with a balanced one. Another objective is raising awareness of the players so that they can better understand the current lifestyles of Bangkok’s millennials. Social media interactions are represented as social media apps and a photo mode.

The social media app in BlueLine focuses on the social feed aspect; it mimics existing social media apps, such as the Facebook, Instagram, and Twitter apps. However, the content of the feed is generated by our design team; none of it is taken from actual sources. To date, numerous studies point toward the impact of social comparison on social media on personal well-being and depressive symptoms [3,60,61]. Regarding this aspect, BlueLine intends to show players through self-monitoring that Blue’s well-being improves after his fixation on social media reduces. This can be achieved through design-generated content (images, comments, and the number of likes) and controlling the time spent on social feed gameplay. Some posts do deal with social comparison to avoid players developing adverse mental health outcomes, such as a decline in psychological well-being [62], and making unfavorable social comparisons [63]; for example, a person may experience a lowering of self-esteem after they see someone showing off their material possessions in a post. In terms of duration, each social media gameplay session has been designed to last <1 minute to prevent players from engaging in heavy social media use and its associated adverse effects, such as self-harm behaviors [64] and a lowering of self-esteem [65].

**Figure 5:**
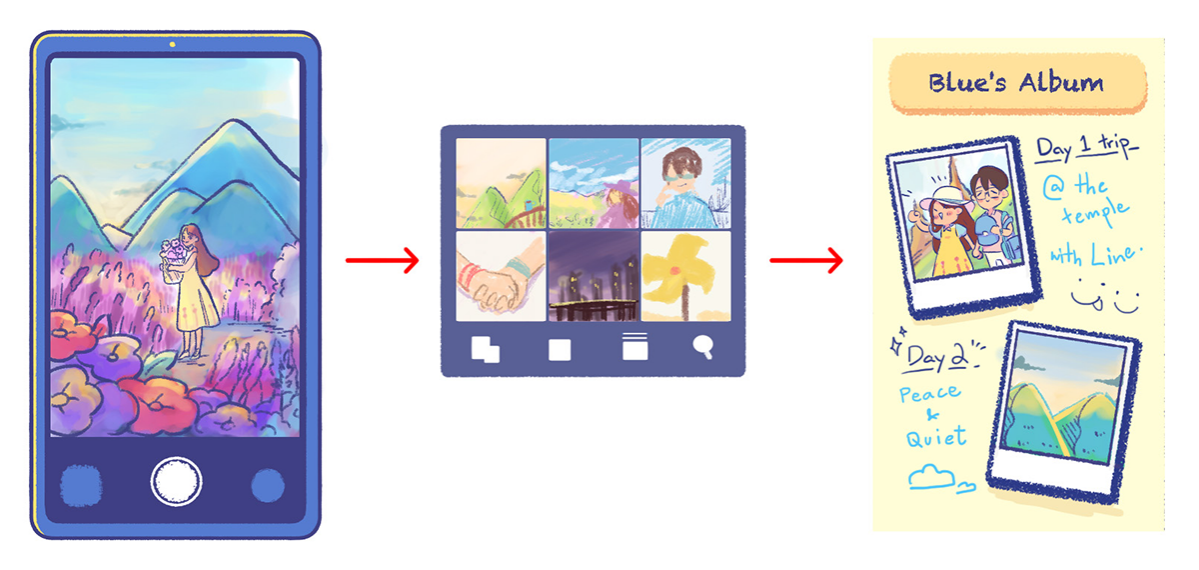
BlueLine’s photo mode.

As many social posts are accompanied by images, the interactive actions in the photo mode will be familiar ground for our target group. Players can use Blue’s phone to take photos of various things, such as food in restaurants and natural scenery (eg, mountains; Figure 5). All the locations presented in BlueLine’s photo mode are replicated from actual destinations in Thailand. This mode can catalyze players to venture outside and engage in relaxation and mindful activities (eg, visiting parks rather than shopping malls).

**Figure 6:**
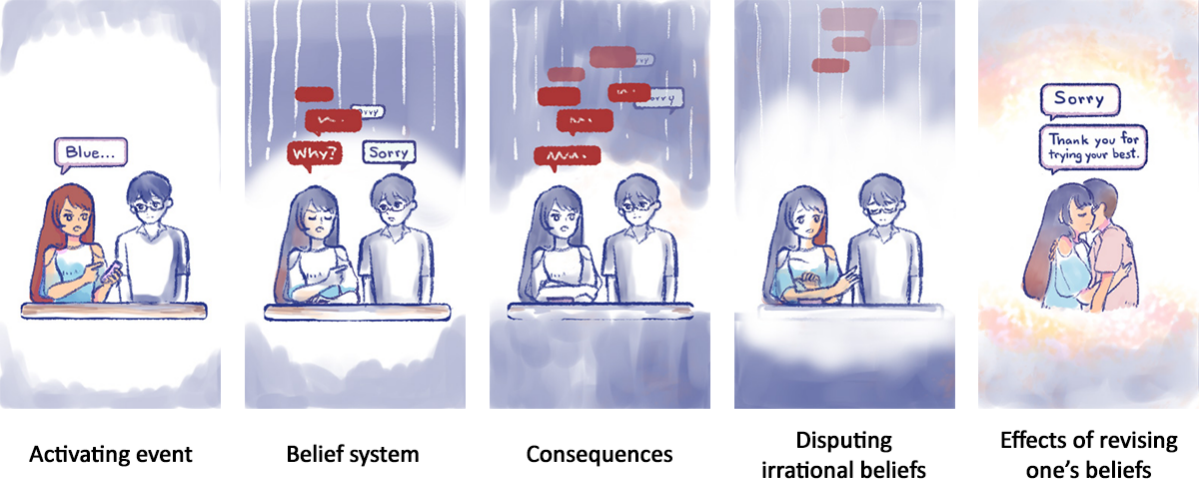
A breakdown of the conversation exchanged between Blue and Line in chapter 9 of BlueLine.

The player’s photos will be saved into a digital photo album that acts as a gratitude journal so that players can look back at such moments and share the photos on the internet. In this album, there will be positive prewritten notations made by Blue to help remind players of the events that occurred in these scenarios (Figure 6). Gratitude reinforcement can improve daily mood and lessen adverse effects (eg, depressive symptoms) [66]. However, multiple studies suggest that combined social comparison and materialism can negatively affect subjective well-being [67-69]. Maintaining a high level of gratefulness can lessen materialistic behaviors [70]. To increase accessibility to Blue’s gratitude journal, we have made it possible for players to access the album via 2 routes: the pause screen and the main menu.

## Discussion

### Principal Findings

This study outlines the process of developing BlueLine, a gamified CBT intervention project on smartphones. BlueLine uses the ABCDE model as the game’s core structure to present the gamified CBT components (Table 1). The core structure integrates the narrative structure before the gameplay with visual elements. The early development is iterative and experimental, and it involves collaboration with CBT specialists in Thailand who have expertise and knowledge in caring for Bangkok’s millennials. Multiple playtests were set up with university students to help with the development process.

As a serious game, BlueLine falls into the visual narrative genre. Its gameplay lasts approximately 80 minutes, broken into 3 acts and 15 chapters. The game is split into short bursts to match a common habit of millennials—resorting to technology for a momentary escape from work—as well as their short attention span [71]. For the context to be meaningful to the target group, the daily routines, behaviors, and life experiences of Blue (the main character) reflect those of Bangkok’s millennials. In addition, interactions in BlueLine are inputted using finger gestures that mimic those involved in the daily use of smartphones.

Primarily, we have designed BlueLine to be used in adjunct with the patient’s primary treatment. It is not designed to tackle depressive symptoms entirely; instead, it focuses on enabling the player to understand the impact of depressive symptoms and learn how to lessen it. In most cases, our target group will not be playing the game while attending a session with their therapist; thus, BlueLine must be able to retain player engagement through gameplay. BlueLine’s gamified CBT mechanics (narrative, verbal interactions, physical interactions, and social media interactions) have been created to be flexible enough to fit into many narrative contexts so that the game encourages repetitions of the following CBT techniques: behavioral activation, self-monitoring, interpersonal skills, positive psychology, relaxation and mindful activities, and problem-solving.

### Strengths

Experience plays a crucial part in this project. Our members have experience making small-scale visual narrative games such as Yumi’s Home [72], a first-person 2D narrative game about depression, familial obligation, and isolation. Furthermore, we have attempted to understand our target players and their behaviors in various ways. First, for 2 years, we were invested in researching the depressive symptoms experienced by Bangkok’s millennials as well as preproduction prototypes. Second, the knowledge and experience of Thai CBT experts and the Department of Mental Health guided our design decisions in the development. There are 4 CBT experts, and each has >10 years of experience in psychotherapy; 2 experts are from the Department of Mental Health and a public psychiatric hospital, and 2 experts are faculty members of psychology departments at Thai universities. Thus, the project can scale up with confidence as we reach production.

To start with, we planned to localize BlueLine in both Thai and English. Those with Thai as their mother tongue can understand the nuances of the language and slang used by the characters, whereas English is meant for the increasingly international population in Bangkok. Players may switch the language at any given time via the options menu.

The story in BlueLine and the characters are based on the standard daily routines of Bangkok’s millennials as well as the real-life stories of development team members. We have identified specific experiences that resonate through the spectrum of millennials’ age range. The gameplay and visuals can be expressed on a deeper level in terms of attention to detail when non–game designers (artists and programmers) also participate in the design process [26].

Depressive symptoms can recur, and that is the takeaway from BlueLine, which shows players through interactions that CBT and therapeutic techniques can reduce the risk of relapse as long as it is maintained [51]. A game such as BlueLine is akin to an empathic listener. To empathize with another person is to understand their feelings, which is a level above mere acknowledgment. Serious games can make player more comfortable reaching out to the CBT-infused gameplay. Finally, BlueLine can enable players to learn by guiding the main character to challenge and overcome their dysfunctional beliefs.

### Limitations

Currently, this project does not accept external funding. The project’s scope is scaled to fit our current workforce, a team consisting of 5 members. Realistically, this is an ideal situation because we are able to maintain complete control over our design decisions. We intend all research and design processes to be free of external monetary influences (eg, investors). Once we complete the project, the app will be released in collaboration with the Department of Mental Health. We will consider a monetization model only after we have completed a randomized controlled trial of BlueLine with Bangkok’s millennials.

Studies have shown the potential of gamification for depression care, but more data are needed to establish the effectiveness of this approach [73,74]. Many factors come into play when considering what gamification needs to accomplish in a serious game; each player may have different depressive symptoms, and the game cannot personalize the experience for each individual. An improper gamified element can have an adverse impact on individuals with mental illnesses [75].

Regarding game addictions, some studies have addressed their adverse effects on an individual’s responsibilities (eg, education [76]) and personality (eg, adverse effects on self-esteem and behaviors [77]). In addition, BlueLine will be played on smartphones, which further extends users’ total screen time. During the scoping phase, we established that the gameplay length is limited to <2 hours and is divided into small chapters to avoid long sessions. In the game, we have designed the interactions to be distinct from nonserious games and promote positive activities such as art therapy.

The BlueLine gameplay length of 1 hour and 20 minutes can be a little problematic, due to factors such as poor concentration, and can result in a higher likelihood of game addiction [44]. When a game loop is long, players can keep playing it repeatedly, which may result in long hours of screen time and addiction. However, BlueLine’s *pause* and *resume* features allow players to take a break and return to the game later. In addition, it is within our scope to implement a feature to remind the player that they have been on the phone playing the game for too long. These features are used in internet-based CBT applications [46] and eHealth programs [47].

Of note, there are practical difficulties in developing mHealth apps in a higher middle–income country such as Thailand because of the lack of existing gamified mHealth apps for benchmarking. The lack of extensive statistical data on Bangkok’s millennials makes it challenging to design specific scenarios in the game. To ensure that our design decisions can lead to the intended experience, we rely on creating multiple iterations of each scenario.

Growing up in Thailand, some of us have noticed that the general public, that is, those who belong to an earlier generation compared with millennials, have negative stereotypical misconceptions of games; they view games as childish activities and believe that a grown-up should not waste time playing games. Moreover, even today, seeking and receiving mental health care is stigmatized in many countries [78] and can lead to social rejection of patients as well as self-rejection experiences [79]. This aspect can hinder the reach of BlueLine to the target group. However, it is worth the attempt, considering the benefits of making CBT accessible to a substantial proportion of Bangkok’s millennials. Thailand is one of many low- and middle-income countries looking at this technology as an opportunity [80].

### Future Directions

In the future, we will collaborate with the Department of Mental Health to conduct a randomized controlled trial of BlueLine with Bangkok’s millennials to assess its efficacy, acceptability, and usability. On the basis of the results, further adjustments will be made before BlueLine and its service avenues are completed.

### Conclusions

In conclusion, the ABCDE model can be the structure of visual narrative games. The gamification of CBT and related therapeutic techniques are interchangeable based on the scenario and its intended experience for the player. To reinforce the same gamified combinations to the player while avoiding monotony in the gameplay, narrative context and visual representation can be used to retain player engagement through the game experience. However, BlueLine’s development process is specifically focused on content related to Thai millennials; thus, its use may be less effective to other groups. BlueLine remains to be investigated in more detail regarding efficacy in adjunctive use with the patient’s existing treatment in Thailand.

## Abbreviations

ABCDE: Activating Events, Beliefs, Consequences, Disputation of Beliefs and Effective New Approaches
CBT: cognitive behavioral therapy
DSM-5: Diagnostic and Statistical Manual of Mental Disorders, Fifth Edition
mHealth: mobile health
NPC: nonplayer character
